# Supplementary material for: Hepatic Polarized Differentiation Promoted the Maturity and Liver Function of Human Embryonic Stem Cell-Derived Hepatocytes via Activating Hippo and AMPK Signaling Pathways
Source: Cells. 2022 Dec 18;11(24):4117. doi: 10.3390/cells11244117 (PMC9776724; doi:10.3390/cells11244117)
Supplement: Supplementary file 1 [file cells-11-04117-s001.zip › cells-2015917-supplementary afterproof-highlight.pdf]

Table S1. Antibodies used in this study.

Items	Species reactivity	Providers	Cat. No.
PE anti-human SOX17 antibody	human	BD Biosciences	561591
APC/Cy7 anti-human CXCR4 antibody	human	BioLegend	306528
Anti-ABCB11 (BSEP) antibody	human	BOSTER	PB9414
Anti-SLC10A1/NTCP1 antibody	human	BOSTER	A06872-1
ZO-1 (D6L1E) Rabbit mAb	human	Cell Signaling Technology	13663
VE-Cadherin (D87F2) XP® Rabbit mAb	human	Cell Signaling Technology	2500
Phospho-YAP (Ser127) (D9W2I) Rabbit mAb	human	Cell Signaling Technology	13008
YAP (D8H1X) XP® Rabbit mAb	human	Cell Signaling Technology	14074
Phospho-AMPKα (Thr172) Antibody	human	Cell Signaling Technology	2531
AMPK Alpha 1 Polyclonal antibody	human	Proteintech	10929-2-AP
GAPDH Antibody	human	Affinity Biosciences	AF7021

Table S2. Primers used in this study.

Gene	Primer sequence (5’-3’)	Accession number
GAPDH	F- GAAGATGGTGATGGGATTTC<br>R- GAAGGTGAAGGTCGGAGTC	NM_001357943.2
BSEP	F- ACATGCTTGCGAGGACCTTTA<br>R- GGAGGTTCGTGCACCAGGTA	XM_017005167.2
MRP2	F- TCTCTCGATACTCTGTGGCAC<br>R- CTGGAATCCGTAGGAGATGAAGA	XM_017015675.3
MDR1	F- GGGATGGTCAGTGTTGATGGA<br>R- GCTATCGTGGTGGCAAACAATA	NM_001348945.2
CTGF	F- CTTGCGAAGCTGACCTGGAAGA<br>R- CCGTCGGTACATACTCCACAGA	NM_001901.4
CYR61	F- GGAAAAGGCAGCTCACTGAAGC<br>R- GGAGATACCAGTTCCACAGGTC	NM_001554.5
CK18	F- TCGCAAATACTGTGGACAATGC<br>R- GCAGTCGTGTGATATTGGTGT	NM_199187.2
CK19	F- AACGGCGAGCTAGAGGTGA<br>R- GGATGGTCGTGTAGTAGTGGC	NM_002276.5
SOX17	F- GGCGCAGCAGAATCCAGA<br>R- CCACGACTTGCCCAGCAT	NM_022454.4
ALB	F- CTGCCTGCCTGTTGCCAAAGC<br>R- GGCAAGGTCCGCCCTGTCATC	NM_000477.7
AFP	F- GGGAGCGGCTGACATTAT<br>R- TGTTTCATCCACCACCAA	NM_001354717.2
HNF4A	F- GGTGTCCATACGCATCCTTGAC<br>R- AGCCGCTTGATCTTCCCTGGAT	XM_047440138.1
G-6-P	F- CTTCTGGACACTGCATGATCACAG<br>R- CCAGTGCAGTCAACCCATAGAAGC	XM_011525474.4
CPS1	F- CTAGCCTGGATTACATGGTCACC<br>R- CCTCAAAGGTACGACCAATAGCC	NM_001122633.3
FXR	F- TGGGGAACTGAA AATGACTC<br>R- ACAGGCAAAGTGTTGAGGAT	XM_011539041.3
CYP7A1	F- ATCCGGACAGCTAAGGAGGATTTC<br>R- TCCCGATCCAAAGGGCATGTAGTA	NM_000780.4
SHP	F- CCCAAGATGCTGTGACCTTTGAG<br>R- TGGGGTCTGTCTGGCAGTTG	XM_011542297.4

Figure S1

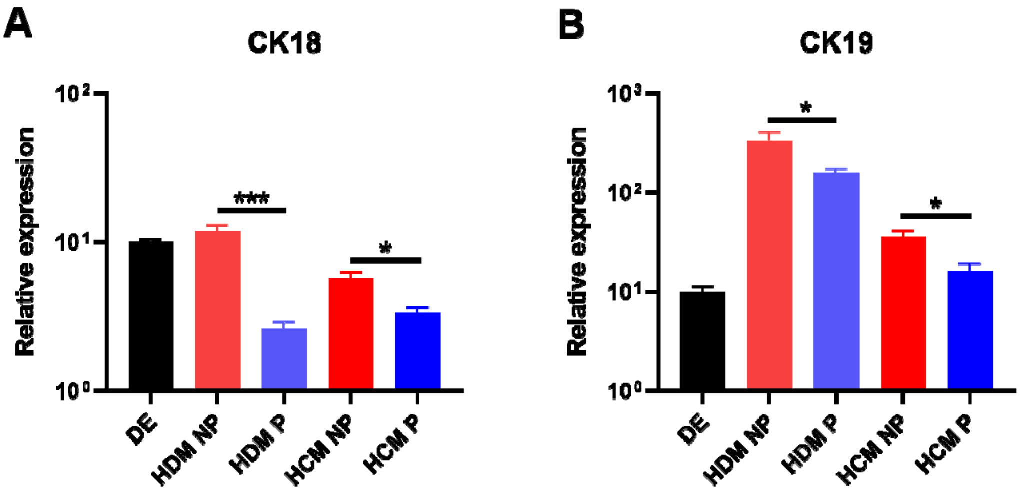

Figure S1. Generation of the polarized hEHs through the transwell-based polarized differentiation

(A,B) Quantitative PCR analysis of CK18 (A) and CK19 (B) in DE cells, the non-polarized hEHs and polarized hEHs cultured with HDM medium, the non-polarized hEHs and polarized hEHs cultured with HCM medium. Aforementioned cells were collected for quantitative PCR analysis at days 7, 21 and 27 during the hepatic differentiation. Relative gene expression represents data normalized to GADPH and calibrated to DE cells. Data represent the mean ± SD.* $p < 0.05$, ** $p < 0.01$, *** $p < 0.001$.

Figure S2

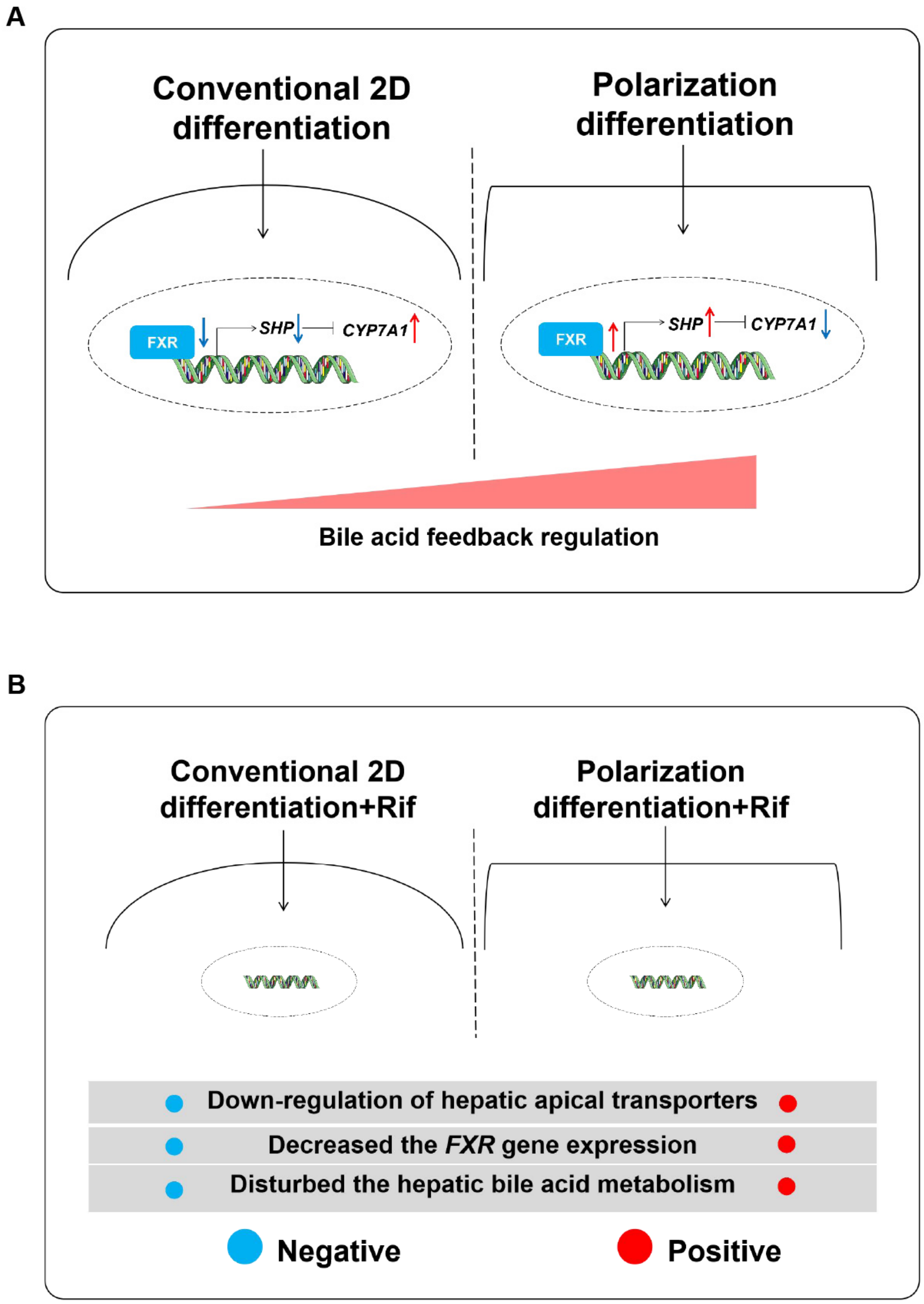

Figure S2. The effects of polarized differentiation on the maturation and function of hEHs. (A) Schematic representation the bile acid feedback regulation in non-polarized and polarized hEHs. (**B)** Schematic representation the polarized hEHs could mimic key phenotypes of dug-induced liver injury similarity to *in vivo* pathological progress.

Figure S3

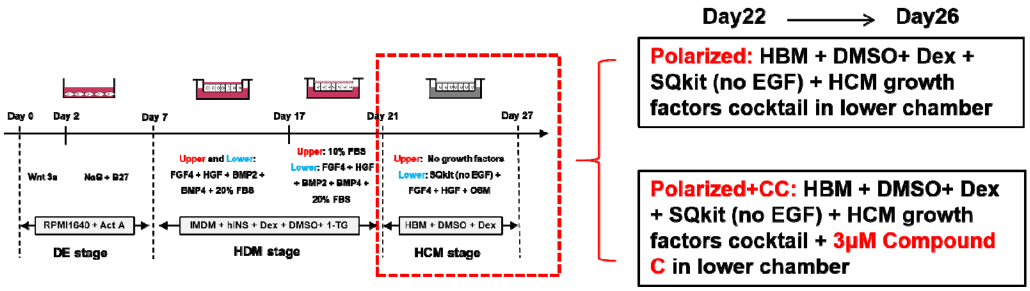

Figure S3. Schematic representation the exprimental strategy for analysis of activation of AMPK signaling pathway invloved in polarity maintenance in polarized hEHs. To verified whether the AMPK signaling pathway mediated the maintenance of the polarity in our polarized hEHs. We collected the polarized hEHs and treated with Compound C (CC), a specific inhibitor of AMPK, for 4 days during the maturation stage with HCM medium culture.
